# Classical scrapie in small ruminants is caused by at least four different prion strains

**DOI:** 10.1186/s13567-021-00929-7

**Published:** 2021-04-15

**Authors:** Alba Marín-Moreno, Patricia Aguilar-Calvo, Juan Carlos Espinosa, María Zamora-Ceballos, José Luis Pitarch, Lorenzo González, Natalia Fernández-Borges, Leonor Orge, Olivier Andréoletti, Romolo Nonno, Juan María Torres

**Affiliations:** 1 Centro de Investigación en Sanidad Animal (CISA-INIA), Valdeolmos, Madrid, Spain; 2 Animal and Plant Health Agency (APHA), Penicuik, Midlothian, UK; 3 Instituto Nacional de Investigação Agrária e Veterinária, Oeiras, Portugal; 4 UMR INRAE ENVT 1225-IHAP, École Nationale Vétérinaire de Toulouse, Toulouse, France; 5 Department of Veterinary Public Health, Nutrition and Food Safety, Istituto Superiore di Sanitá, Rome, Italy; 6 Present Address: Departments of Pathology and Medicine, UC San Diego, La Jolla, CA USA

## Abstract

The diversity of goat scrapie strains in Europe has recently been studied using bioassays in a wide collection of rodent models, resulting in the classification of classical scrapie into four different categories. However, the sole use of the first passage does not lead to isolate adaptation and identification of the strains involved and might therefore lead to misclassification of some scrapie isolates. Therefore, this work reports the complete transmission study of a wide collection of goat transmissible spongiform encephalopathy (TSE) isolates by intracranial inoculation in two transgenic mouse lines overexpressing either small ruminant (TgGoat-ARQ) or bovine (TgBov) PrP^C^. To compare scrapie strains in sheep and goats, sheep scrapie isolates from different European countries were also included in the study. Once the species barrier phenomenon was overcome, an accurate classification of the isolates was attained. Thus, the use of just two rodent models allowed us to fully differentiate at least four different classical scrapie strains in small ruminants and to identify isolates containing mixtures of strains. This work reinforces the idea that classical scrapie in small ruminants is a prion disease caused by multiple different prion strains and not by a single strain, as is the case for epidemic classical bovine spongiform encephalopathy (BSE-C). In addition, the clear dissimilarity between the different scrapie strains and BSE-C does not support the idea that classical scrapie is the origin of epidemic BSE-C.

## Introduction

Prion diseases, or transmissible spongiform encephalopathies (TSEs), are a group of fatal neurodegenerative diseases that affect animals and humans. The group includes several disorders with different origins and epidemiology (sporadic, genetic or acquired), all of which are molecularly based on the conversion of the host-encoded cellular prion protein (PrP^C^) into a disease-associated isoform (PrP^Sc^), which is considered to be the main component of the prion agent [1]. PrP^Sc^ self-catalyses its formation by recruiting PrP^C^ into aggregates that transform PrP^C^ into PrP^Sc^ [2]. TSEs have been reported in animal species in the human food chain, such as small and wild ruminants, bovines [3] and even camels [4]. Indeed, zoonotic transmission is possible, at least in the case of classical bovine spongiform encephalopathy (BSE-C), which is the origin of variant Creutzfeldt-Jakob disease (vCJD) in humans [5] and caused one of the most important food safety crises ever reported.

Scrapie affecting sheep, goats and mouflons [6] has been circulating in Europe for centuries [7] and is exceptionally well transmitted horizontally within herds due to the accumulation of infectious prions in peripheral tissues and biological fluids [8–19] as well as the high persistence of the agent in the environment [20, 21]. Regarding the zoonotic potential of scrapie, some isolates proved to be transmitted to human-PrP transgenic mice showing strain features resembling those of the prion strains causing sporadic Creutzfeldt-Jakob disease (sCJD) in humans [22]. However, in general terms, the zoonotic potential of scrapie is still not fully deciphered [23].

As a consequence of the BSE-C epidemic, active surveillance for TSEs in small ruminants was enhanced in Europe. This increase in active surveillance resulted in the identification of several different disease phenotypes, which points to the existence of different scrapie prion strains [24–29]. Prion strains are thought to be different conformational variants of PrP^Sc^ defined by their biological properties (differences in incubation time, distribution of prion deposits in the brain, and clinical symptoms produced) and biochemical properties when transmitted to congenic hosts [30]. An unusual type of scrapie was reported in 1998 in Norway and named atypical scrapie or Nor-98 [31]. The causative strain is biochemically characterized by a low molecular weight internal proteinase-K resistant PrP^Sc^ (PrP^res^) fragment of 8 kDa [32].

Breeding programmes searching for host prion protein-encoding gene (*PRNP*) variants that confer resistance to scrapie are useful tools to control scrapie in sheep and goat herds. Sheep genotype A_136_R_154_R_171_ is associated with resistance to classical scrapie [33–36], and its promotion within ovine herds resulted in the control of classical scrapie outbreaks [37]. However, atypical scrapie is more common in A_136_R_154_R_171_ and other selected sheep genotypes less susceptible to classical scrapie [38]. For goats, no programmes of breeding for resistance have been implemented to date. However, some goat polymorphisms have been associated with decreased susceptibility to scrapie [39–43].

A previous large and collaborative work investigated the biological properties of goat TSEs in Europe in-depth via the transmission of a collection of goat TSE isolates into different rodent models [44, 45]. This work highlighted that current active surveillance protocols in small ruminants are devoted to discriminating scrapie and BSE-C (leading to the identification of BSE-C in 2 goats [46, 47]) but are poor strategies for identifying the variability of scrapie strains, especially strains of goat scrapie, which is much less frequently investigated than sheep scrapie. Prior biochemical characterization of the goat TSE isolates provided useful information indicating that three different classical scrapie PrP^Sc^ types plus atypical scrapie may exist in goats [44]. Later, a bioassay study identified a range of rodent models useful and reliable for discriminating goat TSEs and BSE. Based on the relative transmission efficiency in the different rodent models and the associated PrP^Sc^ types, four goat classical scrapie types, in addition to atypical scrapie, were identified [45].

The present study further extends the previous knowledge obtained via primary transmission experiments [45] by completing the transmission experiments of the goat TSE isolates in two of the animal models previously reported (TgBov and TgGoat-ARQ). In addition, this study includes several sheep scrapie isolates to extend the analysis to ovine species. The adaptation of the different scrapie isolates in recipient rodents by subpassaging allowed the species barrier to be overcome, thus allowing better discrimination among several scrapie strains and showing that complete differentiation of scrapie strains in small ruminants is feasible by using just two rodent models.

## Materials and methods

### Ethics statement

Animal experiments were carried out in strict accordance with the recommendations in the guidelines of the Code for Methods and Welfare Considerations in Behavioural Research with Animals (Directive 2010/63/EU), and all efforts were made to minimize suffering. Experiments were approved by the Committee on the Ethics of Animal Experiments of the Instituto Nacional de Investigación y Tecnología Agraria y Alimentaria and by the General Directorate of the Madrid Community Government (permit numbers: CEEA 003-2009, CEEA 2011-050, CEEA 2012-002, PROEX 263/15).

### Isolates

The original collection of goat TSE isolates used herein has been previously reported and includes several goat TSE isolates from different geographical origins, and different goat donor PrP genotypes and PrP^Sc^ types [44]. For comparative purposes, one goat BSE-C experimental isolate and eight sheep scrapie isolates from different European countries were included. Further information about the isolates, including their geographical origin, is reported in Table 1. Goat scrapie isolates were provided as 50% macerates in water and immediately frozen at −80 °C until receipt and were later adjusted to 10% weight/volume homogenates in 5% glucose [44, 45]. Sheep isolates were prepared by homogenization of original sheep brains as 10% weight/volume homogenates in 5% glucose. All 10% weight/volume homogenates in 5% glucose were stored at −80 °C prior to inoculation of mice.

**Table 1:** **Goat and sheep TSE isolates employed in this study**.

Country of origin	Species origin	Code (reference)	PrP genotype^a^	PrP^Sc^ type
Italy	Goat	I2 [44, 45]	wt	Classical scrapie
		I3 [44, 45]	240PP	
		I9 [44, 45]	143HR, 240PS	
	Sheep	198/9 [57]	wt	
Netherlands	Goat	N1 [44, 45]	143HR, 240PS	Classical scrapie
		N3 [44, 45]	240PP	
France	Goat	F2 [44, 45]^b^	240PS	Classical scrapie
		F3 [44, 45]	240PP	
		F6 [44, 45]	240PS	
		F16 [44, 45]	240PS	
		gtBSE [44]^c^	211RQ, 240PS	BSE-C
	Sheep	Langlade [57]	wt	Classical scrapie
		PS21 [22]	wt	
		PS09 [22]	wt	
		PS83 [58]	171RR	
Spain	Goat	S2 [44, 45]	240PS	Classical scrapie
		S3 [44, 45]	240PP	
Greece	Goat	G2 [44, 45]	240PP	Classical scrapie
		G3 [44, 45]	143HR, 240PP	
Cyprus	Goat	C1 [44, 45]	240PP	Classical scrapie
		C2 [44, 45]	240PP	
United Kingdom	Goat	UKA2 [44, 45]	240PS	Classical scrapie
		UKB2 [44, 45]	127GS, 240PP	CH1641-like
	Sheep	CH1641 [59]	wt	CH1641-like
Portugal	Sheep	08-8309	141LL	Classical scrapie
		08-27433	wt;141LL	

^a^PrP genotype: “wt” means A_136_R_154_Q_171._ Differences within this haplotype and other *prnp* polymorphisms are indicated

^b^Information for this experimental case. IC inoculation of sheep scrapie PS 48 (ARQ/ARQ sheep)

^c^Information for this experimental case. IC inoculation of classical cattle-BSE 5 (case#139)

### Transgenic mouse lines

Two rodent models were used in this study: TgBov and TgGoat-ARQ. The Bo-Tg110 transgenic mouse line (TgBov) overexpresses bovine PrP at 8-fold the typical PrP expression level in the cow brain in a null mouse PrP background [48–50]. The Go-Tg501 transgenic mouse line (TgGoat-ARQ) overexpresses goat/sheep PrP at 2-fold the typical PrP expression level in the goat brain in a null mouse PrP background [42, 45, 51].

### Bioassay

For this study, the second passage of the abovementioned isolates was performed after transmission into TgBov and TgGoat-ARQ. Thus, the brains of the first-passage animals that were PrP^res^-positive by Western blotting (WB) were pooled and prepared as 10% weight/volume homogenates in 5% glucose to be used as new inocula for the second passage. In some cases, after negative transmission in the first passage, the brains of all the animals were pooled and prepared as mentioned above as new inocula for the second passage, with the exception of the second passage of the N3 isolate in TgBov mice. Inoculation of transgenic mice was performed as previously described [45]. Briefly, groups of 6–9 individually identified mice between 6 and 7 weeks of age were anaesthetized and intracranially inoculated with 20 µL of prion-infected brain homogenate in the right parietal lobe using a 25-gauge disposable hypodermic needle. The neurologic status of the mice was assessed twice a week, and animals were euthanized for ethical reasons when disease progression was evident or at the experimental endpoint [650 days post-inoculation (dpi)]. Necropsy was performed, and the brain was harvested. Half of the brain was collected for histological analysis, and the remaining brain tissue was frozen for PrP^res^ detection by WB. In all cases, survival time and attack rate were calculated for each experiment. The survival time was expressed as the mean survival dpi of all mice scoring positive for PrP^res^, with the corresponding standard deviation. The attack rate was determined as the proportion of mice scoring positive for brain PrP^res^ among the total number of inoculated mice.

### Western blotting procedure

Frozen brain tissues (175 ± 20 mg) were homogenized in 5% glucose in distilled water in grinding tubes (Bio-Rad, Berkeley, USA) adjusted to 10% (w/v) using a TeSeE™ Precess 48™ homogenizer (Bio-Rad) following the manufacturer’s instructions. The presence of PrP^res^ in transgenic mouse brains was determined by WB using the reagents of the commercial ELISA TeSeE kit (Bio-Rad). Brain homogenates (10–100 µL of 10% (w/v) homogenates) were prepared following a previously described protocol [51, 52] in which samples were digested with 40 µg/mL proteinase K (PK) for 15 min at 37 °C and were then loaded in 12% Bis–Tris gels (Criterion XT, Bio-Rad). Proteins were electrophoretically transferred onto PVDF membranes (Millipore, Burlington, USA), which were blocked overnight with 2% BSA blocking buffer. For immunoblotting, membranes were incubated with the Sha31 [53] monoclonal antibody at a concentration of 1 µg/mL. The Sha31 antibody recognizes the 148-YEDRYYRE-155 epitope in the goat PrP^C^ sequence. Immunocomplexes were detected by incubating membranes for 1 h with horseradish peroxidase-conjugated anti-mouse IgG (GE Healthcare Amersham Biosciences. Amersham, UK). Immunoblots were developed with enhanced chemiluminescence ECL Select (GE Healthcare Amersham Biosciences). Images were acquired using a ChemiDoc™ WRS + system and processed using Image Lab 5.2.1 software. All mice used in each inoculation experiment were analysed, but data from representative animals in each experiment were selected for inclusion in the final figures.

### Histological analysis

Brain hemispheres from three to six mice in each inoculation experiment were immediately fixed in neutral buffered 10% formalin (4% 2-formaldehyde) during necropsy, trimmed coronally to obtain the standard areas for the assessment of vacuolar lesion profiles [54] and assigned a random number to allow a double-blind study of the histological and immunohistochemical features. Trimmed tissue specimens were processed routinely, embedded in paraffin, cut to a 4 µm thickness, de-waxed and rehydrated by standard procedures and later embedded in paraffin. After deparaffinization, tissue slices were subjected to different histological assays. Samples were stained with haematoxylin/eosin for evaluation of lesion profiles by standard methods [54]. These methods consisted of semi-quantitative scoring of vacuolation from 0 to 3 in different brain areas to construct the vacuolation profile; the brain areas analysed were the cerebral cortex (CCtx); corpus striatum (Stri); hippocampus (Hpp); thalamus (Thal); hypothalamus (Hpth); midbrain (Midb); cerebellar cortex (CbCtx); and pons/medulla oblongata (PoMe). Within a group of mice, for each brain area, a single value was calculated as the average vacuolation score. In addition, the total vacuolation score for that group was determined by adding the average scores for the different brain areas. The ratios of the average score for each area to the total vacuolation score were expressed as percentages and plotted against the brain areas to produce lesion profiles. In addition, samples were subjected to immunohistochemistry (IHC) using the R486 polyclonal antibody (APHA, Weybridge, UK), which recognizes the 221-CITQYQRESQAYYQR-234 epitope in the goat PrP^C^ sequence, as previously described [55]. Briefly, tissue sections were subjected to antigen retrieval and quenching of peroxidase activity and were later incubated with the R486 polyclonal antibody. Subsequent steps of the immunohistochemical procedure were performed by a commercial immunoperoxidase technique (Vector-Elite ABC kit, Vector Laboratories, San Francisco, USA) according to the manufacturer’s instructions. Finally, sections were counterstained with Mayer’s haematoxylin. The magnitude of PrP^Sc^ accumulation was scored from 0 (absent) to 3 (severe) in the abovementioned brain areas considering the following PrP^Sc^ types: intraneuronal (ITNR), intraglial (ITGL, combined intra-microglial and intra-astrocytic), extracellular glia-associated (GLAS), fine particulate (PRTC), coalescing (COAL), linear (LINR) and plaque (PLAQ, vascular and non-vascular combined). For each PrP^Sc^ type, a single value was calculated as the average of the scores in the different brain areas, and the total brain PrP^Sc^ value was calculated as the sum of the different PrP^Sc^ averages, with a maximum potential score of 21. The average values for each PrP^Sc^ type were converted to percentages with respect to the total PrP^Sc^ content in each mouse, and these percentage values were used for graphical representation of PrP^Sc^ profiles. For IHC in the TgGoat-ARQ model, mouse brains from the first and second passages were used, while in the TgBov model, only mouse brains from the second passage were used. In addition, brain tissue was subjected to a paraffin-embedded tissue (PET) blot protocol as previously described [56] using the Sha31 monoclonal antibody [53].

## Results

This study reports the first and second passages of a wide collection of goat and sheep TSE isolates (Table 1) in the TgBov and TgGoat-ARQ mouse models. Data from the first passage of the goat TSE isolates and some of the sheep isolates have already been published in previous works [44, 45, 49, 57–59]. Brains of PrP^res^-positive TgBov and TgGoat-ARQ animals from the first passage were collected and used to prepare new inocula for use in the abovementioned animal models. Mean survival times and attack rates were analysed for each transmission and compared to the corresponding values obtained in the first passage. In addition, brain PrP^res^ was analysed by WB, and histological analysis of mouse brains was performed.

### Transmission of goat TSE isolates in TgGoat-ARQ mice

As previously observed in the prion field [30] and reported for this subset of goat TSE isolates [44], the efficiency of prion transmission relies on the PrP sequence identity between the donor and host species. Since TgGoat-ARQ mice overexpress small ruminant PrP, the transmission efficiency was fairly high even in the first passage: almost all isolates were transmissible with 100% attack rates, although exceptions were found (i.e., isolate G2) (Table 2) [45]. Animals inoculated with the isolates displayed a wide range of mean survival times, from ~200 dpi to the experimental endpoint (650 dpi). These differences could be representative of the biological properties of the TSE isolates but could also be attributed to the low titre of the inocula or to the presence of different polymorphic variants in the donor goats (Table 1) that might encounter a transmission barrier when transmitted to TgGoat-ARQ mice expressing wild-type goat PrP.

**Table 2:** **Goat and sheep TSE isolate transmission into TgGoat-ARQ and TgBov mice**.

TSE isolate		TgGoat-ARQ				TgBov			
Species	ID	1^st^ passage [45]		2^nd^ passage		1^st^ passage [45]		2^nd^ passage	
		MST ± SD^a^ (n/n_0_)^b^	PrP^res^ signature	MST ± SD^a^ (n/n_0_)^b^	PrP^res^ signature	MST ± SD^a^ (n/n_0_)^b^	PrP^res^ signature	MST ± SD^a^ (n/n_0_)^b^	PrP^res^ signature
Goat	I2	526 ± 123 (6/6)^c^	21 kDa	555 ± 33 (4/4)	21 kDa	453 (1/6)	21 kDa	167 ± 9 (7/7)	21 kDa
	I3	644 ± 14 (4/4)		> 650 (5/5)		464 (1/7)		163 ± 16 (7/7)	
	I9	578 ± 25 (5/5)		547 ± 25 (6/6)		324 ± 90 (6/6)		174 ± 8 (5/5)	
Sheep	198/9	592 ± 13 (6/6)		536 ± 46 (5/5)		259 ± 20 (3/6)		165 ± 7 (7/7)	
Goat	F16	431 ± 22 (3/3)		242 ± 26 (5/5)		348 ± 58 (3/6)		177 ± 9 (6/6)	
	F2	239 ± 21 (4/4)	21 kDa	212 ± 16 (6/6)	21 kDa	343 ± 163 (4/5)	19 kDa	198 ± 13 (6/6)	19 kDa
	F3	287 ± 14 (6/6)		228 ± 7 (6/6)		290 ± 48 (3/6)		191 ± 3 (6/6)	
	F6	468 ± 15 (4/4)		334 ± 6 (6/6)		523 ± 166 (6/6)		178 ± 13 (5/5)	
	N3-fast	451 ± 9 (4/4)		254 ± 50 (6/6)		324^d^, > 650^e^ (2/6)		203 ± 5 (5/5)^d^	
	S2	228 ± 15 (6/6)		233 ± 4 (6/6)		384 ± 149 (6/6)		237 ± 39 (9/9)	
	S3	221 ± 16 (6/6)		233 ± 64 (5/5)^c^		271 ± 19 (6/6)		254 ± 52 (4/4)	
Sheep	PS21	194 ± 5 (6/6)		205 ± 18 (6/6)		244 ± 3 (6/6)		244 ± 3 (6/6)	
	Langlade	> 650 (3/3)		500 ± 23 (4/4)		491 ± 17 (4/6)		229 ± 37 (4/4)	
	PS09	277 ± 31 (5/5)		NA^f^		230 ± 66 (4/6)		168 ± 6 (6/6)	
Goat	UKA2	245 ± 36 (5/5)	21 + 19 kDa	252 ± 8 (6/6)	21 + 19 kDa	255 ± 69 (5/5)	19 kDa	187 ± 6 (6/6)	19 kDa
	UKB2	345 ± 19 (7/7)	19 kDa	214 ± 34 (7/7)	19 kDa	205 ± 12 (7/7)		196 ± 5 (6/6)	
Sheep	08-8309	215 ± 18 (7/7)		NA^f^	–	193 ± 15 (6/6)		168 ± 5 (7/7)	
	08-27433	186 ± 9 (7/7)				187 ± 8 (7/7)		NA^f^	–
	CH1641	217 ± 14 (7/7)				180 ± 3(6/6)			
Goat	G2	> 650 (1/4)	21 kDa	470 ± 22 (7/7)	21 kDa	610 (1/6)	19 kDa	348 ± 85 (6/6)	19 kDa
	G3	466 ± 35 (4/4)		375 ± 25 (4/4)		> 650 (0/6)	–	> 650 (0/3)	–
	N1	437 ± 4 (4/4)		339 ± 19 (5/5)		649 ± 9 (3/3)	19 kDa	495 ± 48 (4/4)	19 kDa
	N3-slow	451 ± 9 (4/4)		254 ± 50 (6/6)		324, > 650 (2/6)		493 ± 72 (6/6)^e^	
	C1	483 ± 15 (4/4)		301 ± 10 (4/4)		650 (2/2)		371 ± 51 (6/6)	
	C2	475 ± 31 (5/5)		324 ± 9 (4/4)		> 650 (2/6)		387 ± 12 (3/3)	
Sheep	PS83	302 ± 18 (6/6)		NA^f^	–	> 650 (1/10)		542 ± 50 (5/5)	
Goat	gtBSE	346 ± 16 (6/6)	BSE	309 ± 19 (5/5)	BSE	260 ± 14 (6/6)	BSE	266 ± 17 (5/5)	BSE

^a^Mean survival times in days post-inoculation ± standard deviation

^b^Attack rate, where “n” is the number of PrP^res^-positive animals as determined by WB and “n_0_” is the total number of inoculated animals

^c^Some animals died earlier than the rest of their cagemates, probably due to concomitant disease, but were found to be PrP^res^-positive

^d^Named N3-fast

^e^Named N3-slow

^f^Not available

These phenomena were abolished on second passage. As expected, all isolates were again transmissible, showing high attack rates (Table 2). Some isolates (i.e., all the Italian isolates) maintained their association with long mean survival times, indicating that those values are intrinsic characteristics of the goat TSE inocula. However, animals inoculated with some other isolates (i.e., the Dutch and Greek isolates, F6 and F16) exhibited reduced mean survival times on the second passage, thus suggesting that a transmission barrier was overcome in the second passage or that these inocula had truly low infectious titres.

**Figure 1:**
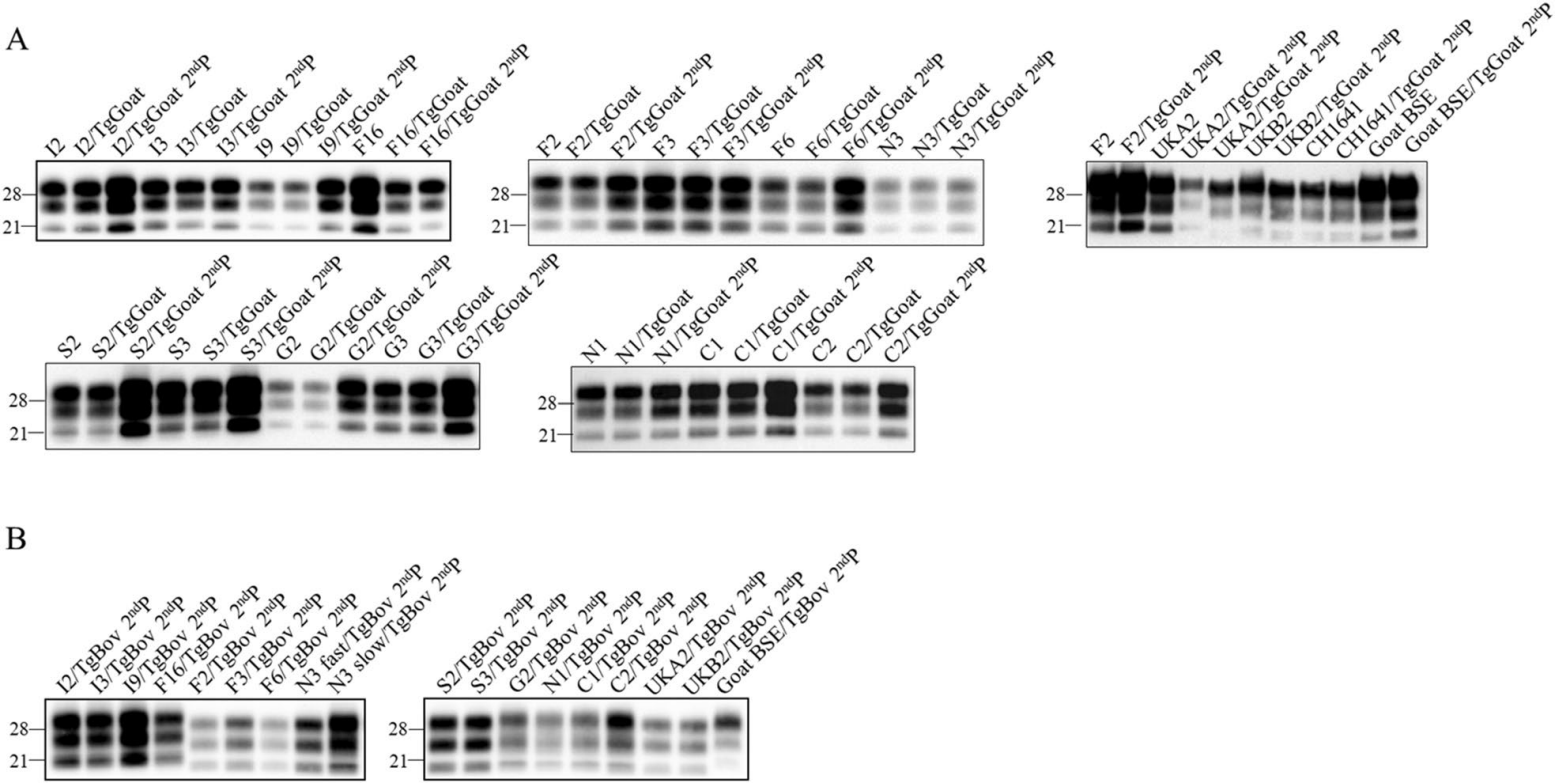
**Brain PrP**^**res**^** in TgGoat-ARQ and TgBov mice after the second passage of goat TSE isolates.** The biochemical profile of brain PrP^res^ after the first and second passages of goat isolates in TgGoat-ARQ (**A**) and TgBov (**B**) mice was compared to that of the original inoculum by WB using the Sha31 monoclonal antibody. Molecular weight markers in kDa are included.

The brains of all inoculated animals were analysed by WB to assess the presence of PrP^res^ and its main characteristics. Western blot analysis of the brains of the animals inoculated in the first passage showed two main PrP^res^ signatures (Table 2; Figure 1A) [45], with most of the isolates producing the classical three-band pattern (corresponding to the different glycosylation states of PrP) characterized by the 21 kilodalton (kDa) molecular weight of the non-glycosylated band. However, the goat TSE isolate UKB2 produced a different PrP^res^ signature characterized by double non-glycosylated bands with a lower molecular weight, approximately 19 kDa. The goat isolate UKA2 produced both PrP^res^ signatures, with some animals (2 of 5) showing the classical 21 kDa signature and other animals (3 of 5) showing the 19 kDa signature (Table 2; Figure 1A) [45].

Analysis of the PrP^res^ signature obtained in the second passage showed no differences from that obtained in the first passage (Table 2; Figure 1A). In the case of UKA2, both the 21 (3 of 6 animals) and 19 kDa (3 of 6 animals) PrP^res^ signatures were still observed in individual animals in the second passage. Consistent with these results, UKB2-inoculated mice in the second passage presented the 19 kDa PrP^res^ signature. The rest of the isolates invariably produced just the 21 kDa PrP^res^ signature. Both the 21 and 19 kDa PrP^res^ signatures shown by the classical scrapie isolates remained different and clearly distinguishable from those of the goat BSE-C isolate included for comparison (Figure 1A).

### Transmission of goat TSE isolates in TgBov mice

In the first passage already published [45] and included here for comparative purposes, TgBov mice were a useful tool to highlight differences between the different goat TSE inocula (Table 2). In these transmissions, the donor and host PrP belong to different species; thus, a species barrier was expected. Variable attack rates ranging from 100% (isolates N1, I9, F6, S2, S3, C1, UKA2 and UKB2) to other intermediate values (I2, I3, N3, F2, F3, F16 and C2) to 0% (isolate G3) were observed. The mean survival times ranged from ~200 dpi to the endpoint of the experiment (650 dpi).

In the second passage, once the species barrier was overcome, more information was obtained for the different inocula (Table 2). The Italian isolates displayed 100% attack rates combined with short incubation times of approximately 170 dpi. Regarding the rest of the inocula, isolate G3 remained non-transmissible in the second passage, while the attack rates of the rest of the inocula (isolates N3, F3, F16 and C2) generally increased. Overall, the mean survival times were shortened in animals inoculated with all isolates, with almost all times under 400 dpi.

It is worth noting that the first passage of the N3 isolate in TgBov resulted in just two positive animals that showed very dissimilar mean survival times: 324 dpi and 703 dpi (Table 2). This difference prompted us to perform a second passage with inocula from both of these animals. The N3-fast inoculum was derived from the brain of the animal with the 324 dpi survival time, while the N3-slow isolate was obtained from the animal with the 703 dpi survival time. The second passage of these isolates resulted in the isolation of two different prion agents (Table 2). N3-fast was characterized by a short incubation time of ~200 dpi, while N3-slow exhibited a longer incubation time of ~500 dpi.

WB analysis of brain PrP^res^ from the inoculated TgBov mice had very interesting results in the first passage (Table 2; Figure 1B) [45]. The Italian isolates and F16 were the only isolates producing the 21 kDa PrP^res^ signature, while the rest of the transmissible inocula generated the 19 kDa signature. In the second passage, all Italian isolates and the F16 isolate faithfully reproduced the 21 kDa PrP^res^ signature (Table 2; Figure 1B). The rest of the isolates produced the 19 kDa signature (Table 2; Figure 1B). Both the 21 and 19 kDa PrP^res^ signature shown by the classical scrapie isolates remained different and clearly distinguishable from those of the goat classical BSE isolate included for comparison purposes (Figure 1B).

### Transmission of sheep TSE isolates in the TgGoat-ARQ and TgBov models

As was already observed for the goat TSE isolates, the sheep TSE isolates were transmitted with high efficiency in TgGoat-ARQ mice, with all isolates showing 100% attack rates (Table 2). The incubation times ranged from ~200 dpi to the experimental endpoint. Although a second passage was not available for all isolates, the fast isolates in the first passage (scrapie CH1641, PS09, PS83, 08-8309 and 08-27433) were expected to maintain their association with short survival times in the second passage, as observed for PS21 (Table 2). For the slow isolates in the first passage (198-9 and Langlade), the mean survival time was slightly reduced but still greater than 500 dpi. As expected, all isolates were again transmissible, showing high attack rates.

**Figure 2:**
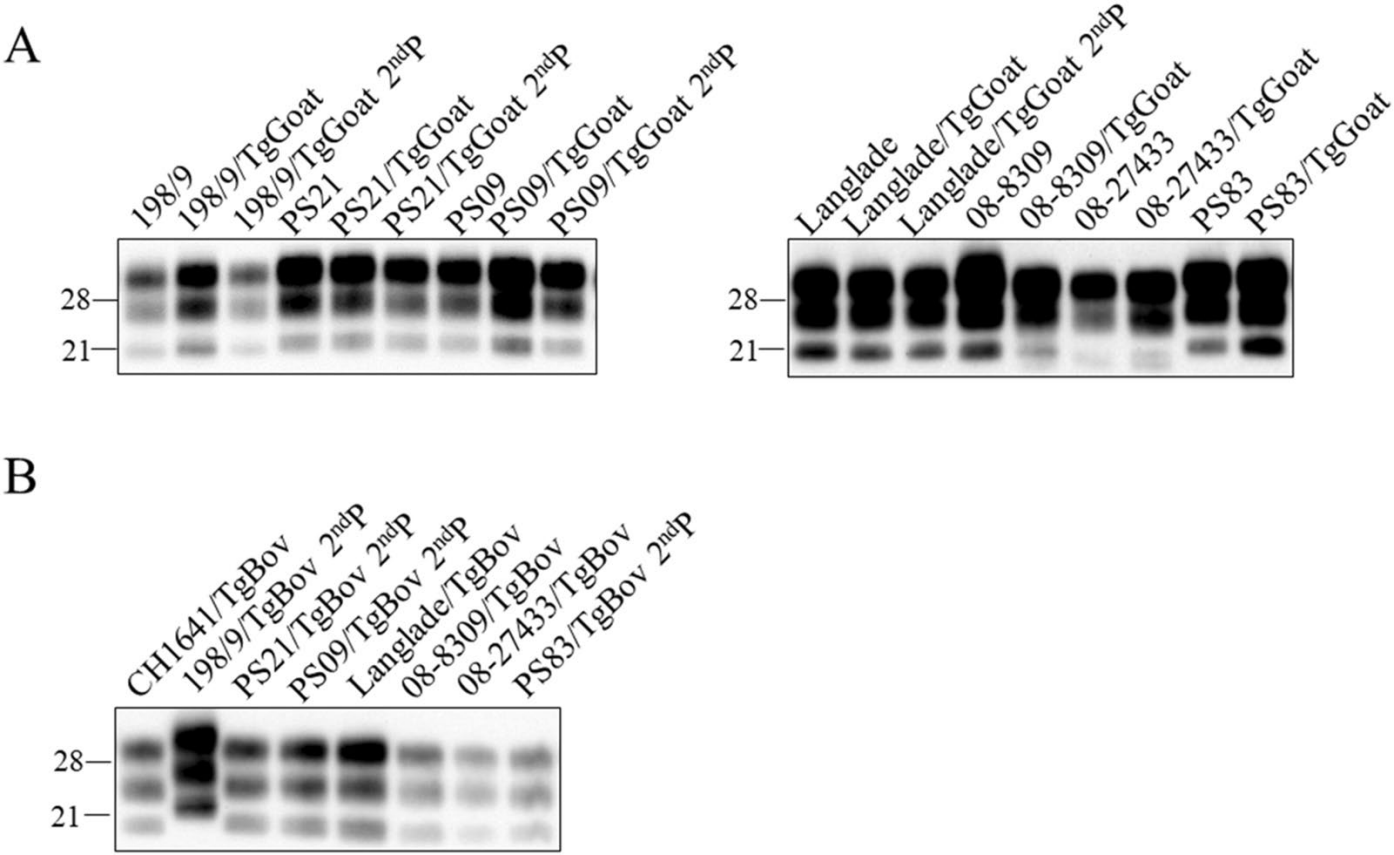
**Brain PrP**^**res**^** in TgGoat-ARQ (A) and TgBov (B) mice after the second passage of sheep TSE isolates.** The biochemical profile of brain PrP^res^ after the first and second passages of sheep isolates in TgGoat-ARQ (**A**) and TgBov (**B**) mice was compared to that of the original inoculum by WB using the Sha31 monoclonal antibody. Molecular weight markers in kDa are included.

WB analysis of the brains of the inoculated TgGoat-ARQ mice in the first passage showed the same two PrP^res^ signatures found for goat TSE isolates after passage in TgGoat-ARQ mice (Table 2) [45]. The Italian and French isolates produced the 21 kDa signature, whereas the Portuguese isolates 08-8309 and 08-27433 and sheep scrapie CH1641 produced the double 19 kDa PrP^res^ signature (Table 2; Figure 2A). The second passage showed no differences from the first passage (Table 2; Figure 4A).

In the first passage in TgBov mice, sheep TSE isolates produced different attack rates depending on the inoculum (Table 2). French PS21, sheep scrapie CH1641 and the Portuguese isolates exhibited 100% attack rates, while the Italian isolate 198-9 and the French isolates Langlade and PS09 exhibited intermediate attack rates of ~50%. Finally, the French isolate PS83 showed a low attack rate of 10%. Mean survival times were generally short, <300 dpi even in animals inoculated with isolates with an intermediate attack rate, with the exception of the PS83 isolate, which exhibited an extremely long incubation time exceeding the experimental endpoint (Table 2). In the second passage and after the species barrier was overcome, all isolates displayed 100% attack rates accompanied by extremely short incubation times, with the exception of isolate PS83, which remained a slow isolate associated with a mean survival time greater than 500 dpi (Table 2).

WB analysis of the brain PrP^res^ in the inoculated TgBov mice was consistent with the results obtained for the goat isolates. The Italian sheep isolate produced a 21 kDa PrP^res^ signature (Figure 2B). The rest of inocula generated a 19 kDa signature. In the second passage, the same results were obtained (Figure 2B).

### Categorization of the goat TSE isolates according to biological and biochemical properties

In a previous work reporting the first passage of these inocula in a wide variety of rodent models [45], the same goat TSE isolates used here were classified into different categories by their comparative transmission in all models using a numerical parameter called the transmission efficiency (TE). This parameter combines both the attack rate and mean survival time to offer a complete picture of the transmission efficiency of a certain isolate in a specific rodent model and is very useful for isolate classification when graphically represented [45]. The brain PrP^res^ signature is also useful for classification [45].

**Figure 3:**
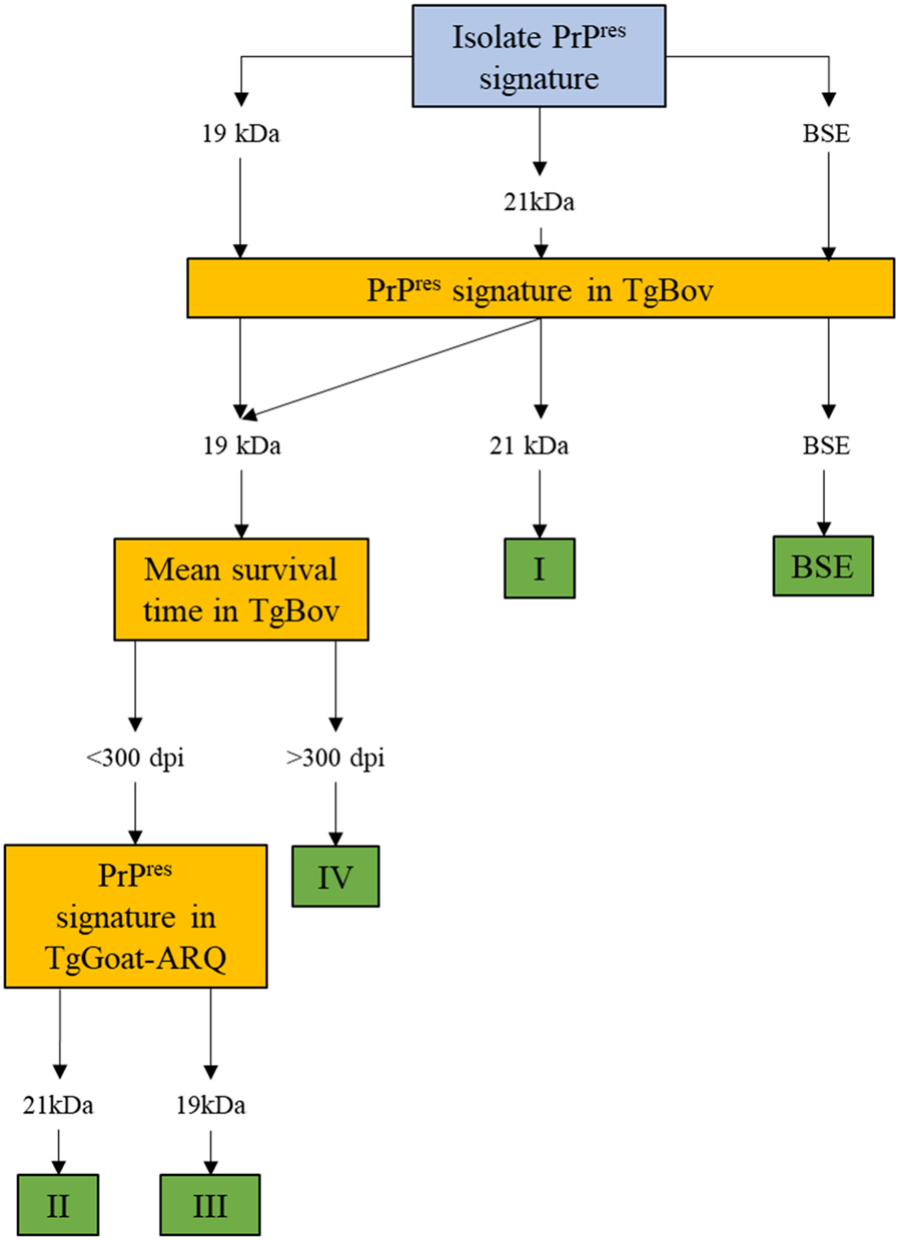
**Decision tree for classical scrapie classification using second-passage results in TgGoat-ARQ and TgBov mice**.

In the present study, we report the second passages of just two of the previously investigated rodent models. Each second passage transmission was qualitatively named fast or slow depending on the mean survival time of the inoculated animals: isolates leading to a mean survival time of greater than 300 dpi were classified as slow, while those leading to a mean survival time of less than 300 dpi were classified as fast (Table 3; Figure 3). The combination of this qualitative classification of survival time with the PrP^res^ signature allowed us to re-configure the four categories proposed in the previous study [45] and to extend this categorization to sheep TSE isolates. Category I isolates are slow in TgGoat-ARQ mice but fast in TgBov mice and show a 21 kDa non-glycosylated signature in both mouse models (21 kDa signature and slow in TgGoat-ARQ mice + 21 kDa signature and fast in TgBov mice). The production of the 21 kDa signature in TgBov mice makes these isolates readily distinguishable from the rest of the isolates (Figure 3). Category I includes the Italian isolates (both goat and sheep). The F16 isolate shares common features with this category. Category II isolates are characterized by the 21 kDa signature and a classification as fast in TgGoat-ARQ mice and a 19 kDa signature and a classification as fast in TgBov mice; these isolates include the goat isolates F2, F3, F6, N3-fast, S2 and S3 as well as the sheep isolates Langlade, PS21 and PS09. Category III includes goat UKB2, and the sheep isolates 08-8308 and 08-27433 from Portugal as well as sheep scrapie CH1641 (double 19 kDa signature and fast in TgGoat-ARQ mice + 19 kDa signature and fast in TgBov mice). Categories II and III share the same transmission features in both models, with the exception of the PrP^res^ signature in TgGoat-ARQ mice; transmission in these mice is thus indispensable for differentiation between these categories (Figure 3). The goat UKA2 isolate shares common features with both categories II and III, since its passage in TgGoat-ARQ mice can produce either the 21 kDa or 19 kDa PrP^res^ signature. Category IV includes the goat isolates G2, G3, N1, N3-slow, C1 and C2 and possibly sheep scrapie PS83 (21 kDa signature and slow in TgGoat-ARQ mice + 19 kDa signature and slow in TgBov mice). The slow incubation times of these isolates, particularly in TgBov mice, differentiates them from the rest of the isolates (Figure 3).

**Table 3:** **Classical scrapie classification of the isolates included in this study**.

Category	Isolate ID (origin species)	Characteristics in TgGoat-ARQ		Characteristics in TgBov	
		Mean survival time^a^	PrP^res^ signature	Mean survival time^a^	PrP^res^ signature
I^b^	I2 (Goat)	Slow	21 kDa	Fast	21 kDa
	I3 (Goat)				
	I9 (Goat)				
	198-9 (Sheep)				
II^c^	F2 (Goat)	Fast	21 kDa	Fast	19 kDa
	F3 (Goat)				
	F6 (Goat)				
	N3-fast (Goat)				
	S2 (Goat)				
	S3 (Goat)				
	PS21 (Sheep)				
	Langlade (Sheep)				
	PS09 (Sheep)				
III^c^	UKB2 (Goat)	Fast	19 kDa	Fast	
	08-8309 (Sheep)				
	08-27433 (Sheep)				
	CH1641 (Sheep)				
IV	G2 (Goat)	Slow	21 kDa	Slow	19 kDa
	G3 (Goat)				
	N1 (Goat)				
	N3-slow (Goat)				
	C1 (Goat)				
	C2 (Goat)				
	PS83 (Sheep)				

^a^Mean survival times greater than 300 dpi were considered slow, while mean survival times less than 300 dpi were considered fast

^b^Goat isolate F16 showed some features compatible with classification in group I, but more data are needed to further determine whether one or more strains are present in this isolate

^c^Goat isolate UKA2 showed some features compatible with classification in groups II and III, but more data are needed to further determine whether one or more strains are present in this isolate

### Histological analysis

Histopathological studies of isolates classified in the same group showed similar lesion profiles and PrP^Sc^ deposition patterns by IHC and PET blotting.

**Figure 4:**
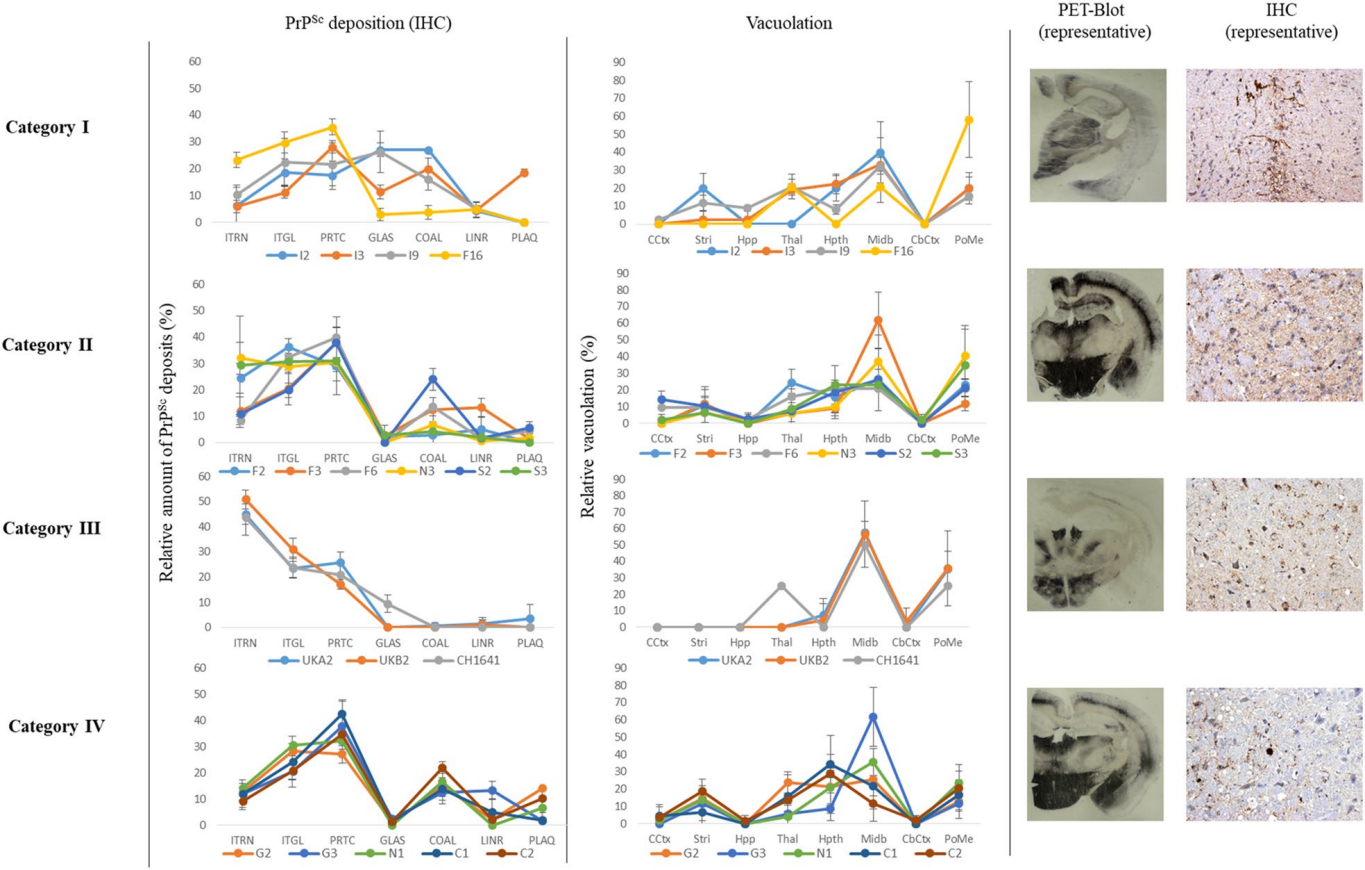
**Histological analysis of goat TSE isolate transmission in TgGoat-ARQ mice.** IHC for PrP^Sc^ was performed using the R468 polyclonal antibody. PET blotting was performed using the Sha31 monoclonal antibody. ITNR: Intraneuronal; ITGL: Intra-microglial and intra-astrocytic; PRTC: fine particulate; GLAS: extracellular glia-associated; COAL: Coalescing; LINR: Linear; PLAQ: plaques (vascular and non-vascular); CCtx: Cerebral cortex; Stri: Striatum; Hpp; Hippocampus; Thal: Thalamus; Hpth: Hypothalamus; Midb: Midbrain; CbCtx: Cerebellar cortex; PoMe: Pons/medulla oblongata.

Histological analysis in TgGoat-ARQ mice inoculated with the goat TSE isolates showed no clear differences in lesion profiles and PrP^Sc^ distribution patterns in animals inoculated with the Italian, French and Spanish isolates; these patterns were characterized by vacuolation, especially in the midbrain and pons/medulla oblongata, as well as by mainly intraneuronal, intraglial and fine particulate PrP^Sc^ deposits (Figure 4). However, PET blot images reflected clear differences between the group containing the Italian isolates and the F16 French isolate and the group containing the Spanish isolates and the remaining French isolates (Figure 4). By contrast, mice inoculated with the Dutch, Greek and Cypriot isolates showed more intense vacuolation in the hypothalamus and thalamus as well as coalescing and fine particulate deposits of PrP^Sc^ (Figure 4), while mice inoculated with the British isolates showed increased intraneuronal, intraglial and particulate PrP^Sc^ deposits combined with vacuolation restricted to the midbrain and pons/medulla oblongata. PET blot images corresponding to these isolates also showed clear differences between these isolates and the rest of the goat inocula included in the study (Figure 4).

**Figure 5:**
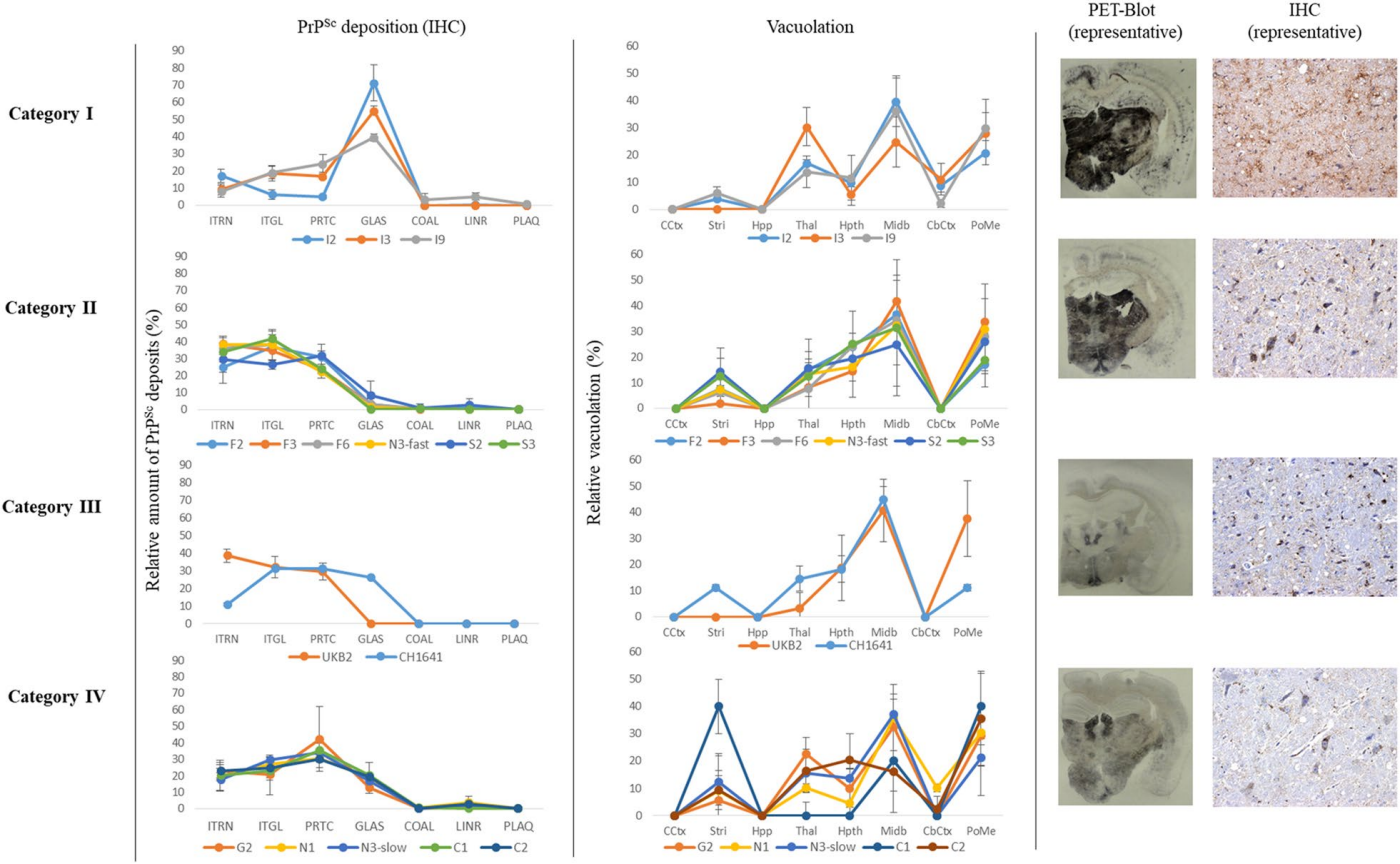
**Histological analysis of goat TSE isolate transmission in TgBov mice.** IHC for PrP^Sc^ was performed using the R468 polyclonal antibody. PET blotting was performed using the Sha31 monoclonal antibody. ITNR: Intraneuronal; ITGL: Intra-microglial and intra-astrocytic; PRTC: fine particulate; GLAS: extracellular glia-associated; COAL: Coalescing; LINR: Linear; PLAQ: plaques (vascular and non-vascular); CCtx: Cerebral cortex; Stri: Striatum; Hpp; Hippocampus; Thal: Thalamus; Hpth: Hypothalamus; Midb: Midbrain; CbCtx: Cerebellar cortex; PoMe: Pons/medulla oblongata.

Histological analysis in TgBov mice inoculated with goat TSE isolates also allowed the identification of different neuropathological patterns. TgBov mice inoculated with the Italian isolates were easily differentiated from all other mice by the vacuolation in the thalamus as well as the presence of glia-associated PrP^Sc^ deposits, which were almost completely absent in mice inoculated with the rest of the inocula (Figure 5). Mice inoculated with the other isolates showed similar vacuolation patterns mainly characterized by severe vacuolation in the midbrain and pons/medulla oblongata and less severe vacuolation in the thalamus, hypothalamus and striatum (Figure 5). The PrP^Sc^ deposits detected were mainly intraneuronal, intraglial and fine punctuate (Figure 5). PET blot images corresponding to these isolates also showed clear differences between these isolates and the Italian isolates (Figure 5).

For sheep isolates, only TgBov and TgGoat-ARQ mice after the first passage of CH1641 were available for histological analysis. The lesion profile and PrP^Sc^ distribution observed for these isolates were similar to those observed for the goat isolates UKA2 and UKB2. PET blot images also confirmed that these isolates resulted in similar neuropathological patterns (Figures 2, 3), suggesting the isolation of the same scrapie strain.

## Discussion

This work reports the complete transmission study of a wide collection of sheep and goat TSE isolates in two rodent models: TgGoat-ARQ and TgBov. Importantly, all goat isolates included in this work were previously biochemically characterized and later transmitted into a larger collection of seven rodent models, including RIII mice, bank voles and other transgenic mouse lines overexpressing ovine, bovine and wild-type mouse PrP [44, 45]. Both studies allowed an initial classification of the goat isolates into several categories due to the intrinsic characteristics of the inocula as well as their transmission properties when inoculated into the different rodents, suggesting the existence of several goat classical scrapie strains plus atypical scrapie circulating in Europe [44, 45]. In the present study, sheep isolates were also included to investigate whether the categories established for goat isolates could be extended to sheep scrapie isolates.

Although informative, primary passage of a prion isolate in one or several hosts may not reveal all the characteristics of a certain prion inoculum due to the species barrier phenomenon [30]. Amino acid differences between the donor and host PrP may result in inefficient prion propagation in the first passage. Typically, as the difference between the donor and host PrP is eliminated, the second passage results in increased attack rates and decreased mean survival times, due to complete adaptation of the prion agent to the host PrP sequence. Regarding this concrete study, many goat isolates exhibited partial attack rates in the first passage when transmitted into heterologous PrP rodent models (wild-type mice, bank voles and TgBov mice). However, this phenomenon also occurred with some isolates transmitted into homologous models (TgGoat-ARQ mice), possibly due to the presence of polymorphic variants in the *PRNP* gene of the donor goats [45]. As a consequence of this phenomenon, classification of the isolates after adaptation in two rodent models allowed a new classification of scrapie strains. Thus, the second passage overcame the species barrier and provided new information about some of the prion isolates. Interestingly, in the case of the N3 isolate, a second passage in TgBov mice allowed the isolation of two different strains derived from the same inoculum. Whether these two strains co-existed in the original material and were then selected for by passage in TgBov mice (strain selection) or whether one of them was later generated as a consequence of passage in TgBov mice (strain adaptation) is not known. Isolates S2 and S3 were originally grouped with UKA2 and UKB2 by their TE profile in several rodent models [45], but the second passage proved that these isolates had a better fit in category II, leaving UKA2 and UKB2 plus the Portuguese isolates and scrapie CH1641 from sheep categorized alone due to their unique PrP^res^ signature determined by WB (19 kDa in all or some inoculated TgGoat-ARQ mice). The scrapie isolates in this group may contain strain mixtures containing different proportions of scrapie strains producing the 21 kDa and 19 kDa signatures. Indeed, the UKA2 isolate clearly harbours a high level of the 21 kDa component present in the original goat PrP^res^ and partially reproduced in TgGoat-ARQ mice, while UKB2, with the 19 kDa PrP^res^ in the original inoculum, was more similar to CH1641 and did not produce the band associated with the 21 kDa PrP^res^ in any of the TgGoat-ARQ mice. To definitively prove the existence of two different strains in the UKA2 isolate, separate second passages of the animal isolates producing the 21 kDa and 19 kDa signatures are currently ongoing.

In addition, this work highlights that extending the analysis of goat TSEs by performing the second passage of the inocula in just two rodent models, one heterologous (TgBov mice) and one homologous (TgGoat-ARQ mice), is enough to classify the isolates into different categories. Both models are also very different with regard to brain PrP^C^ expression level. While TgGoat-ARQ mice express brain PrP^C^ at a level 2-fold higher than that in the goat brain [42, 45, 51], TgBov mice express brain PrP^C^ at a level 8-fold higher than that in the cow brain [48–50]. It has been reported that different levels of prion protein expression in the host can modify the features of the transmitted prion strains [60]. This phenomenon was already noted in a report of the first passage of goat TSE isolates in ovine PrP transgenic mice with different brain PrP^C^ expression levels; divergent results in terms of prion strain features were obtained [45]. It is possible that strain shift may occur in TgBov model mice due to their high PrP expression levels. This possibility should be investigated by transmission of the isolates in other bovine PrP transgenic mice with lower brain PrP expression levels. However, the absence of divergent PrP^res^ profiles among the animals and the uniformity of the incubation times after two passages in TgBov mice argue against the occurrence of mutation events attributable to PrP^C^ overexpression in our study. The main categories identified in previous work after the first passage [45] were maintained with only slight differences due to re-assignment of certain isolates to other groups considering the new classification parameters. Moreover, the categories were maintained in both small ruminant species, showing that the same strains can be found in both species. Therefore, scrapie bioassays in the specific combination of TgGoat-ARQ plus TgBov mice seems to be a robust and high-confidence approach for in-depth scrapie strain typing. Transmission into TgBov mice, in a heterologous PrP context, was highly informative, allowing the discrimination of three of the four classical scrapie categories detailed in this study (Figure 5). This result prompted us to build a decision tree to guide strain categorization. As a first step, the PrP^res^ signature in TgBov mice allows clear identification of the isolates in category I due to their characteristic 21 kDa PrP^res^ signature. Then, among isolates producing the 19 kDa PrP^res^ signature, the mean survival time in TgBov mice differentiates isolates with slow incubation and the 19 kDa PrP^res^ signature as belonging to category IV, while a fast survival time and production of the 19 kDa PrP^res^ signature identify isolates in categories II and III. To discriminate between these two categories, homologous transmission in TgGoat-ARQ mice is necessary, leading to the differentiation of the fast 21 kDa PrP^res^-producing isolates in category II from the fast 19/21 kDa PrP^res^-producing isolates in category III (Figure 5).

Our qualitative system (Table 3) allowed us to perform an initial classification of the classical scrapie isolates that was consistent with the report of the first passage of goat TSE isolates in seven rodent models [45]: Italian isolates (category I) vs non-Italian isolates (all other categories). All inocula from Italy, either of sheep or goat origin, showed uniform behaviour in both TgGoat-ARQ and TgBov mice, which was mainly characterized by the 21 kDa PrP^res^ signature on Western blots and an extremely short incubation time after transmission into TgBov mice. The French isolate F16 also exhibited these features when transmitted into TgBov mice, suggesting its classification into category I, but its incubation was faster than that of the Italian isolates in TgGoat-ARQ mice. This different behaviour in TgGoat-ARQ mice may suggest the existence of a strain mixture in this particular isolate. However, more experiments are needed to determine the definite strain composition of this particular isolate. For the rest of the goat scrapie isolates, one main distinction can be derived from the incubation times in the two models: fast–fast (categories II and III) vs slow-slow (category IV). Finally, those isolates characterized by fast–fast behaviour in both models can be further differentiated by their production of the 21 kDa (category II) or the 19 kDa (category III) PrP^res^ signature in TgGoat-ARQ mice. However, other important distinctions must be made for goat isolates such as UKA2 (category III), since in WB, these isolates can produce either the 19 kDa PrP^res^ signature or the 21 kDa signature. This finding suggests the existence of a strain mixture in this isolate due to the emergence of scrapie producing the 19 kDa signature reminiscent of the experimental CH1641 isolate, as this finding was already reported after the first passage [45], and the rest of the isolates were included in category III (goat UKB2 and sheep isolates 08-8309 and 08-27433).

As a consequence of this classification, we suggest the existence of at least four different classical scrapie strains circulating in Europe: (I) the strain with a 21 kDa-slow profile in TgGoat-ARQ mice and a 21 kDa-fast profile in TgBov mice (detected in all Italian isolates and the F16 French isolate); (II) the strain with a 21 kDa-fast profile in TgGoat-ARQ mice and a 19 kDa-fast profile in TgBov mice; (III) the strain with a 19 kDa-fast profile in TgGoat-ARQ mice and a 19 kDa-fast profile in TgBov mice, similar to CH1641; and (IV) the strain with a 21 kDa-slow profile in TgGoat-ARQ mice and a 19 kDa-slow profile in TgBov mice. Mixtures of these strains can also be found in a single isolate.

In summary, this work rejects the idea of considering classical scrapie as a uniform prion disease caused by a single strain, as is the case for epidemic BSE-C. Moreover, the clear differences in the biological and biochemical properties among the four classical scrapie strains identified in this work and the BSE isolate included for comparative purposes (Table 2; Figure 3) indicate that none of the classical scrapie strains that are currently circulating in Europe are the origin strain of epidemic BSE-C (Figure 3). This view has already been suggested for scrapie in sheep [24–29], but the variability of scrapie in goats has not been investigated. With at least four different classical scrapie strains plus atypical/Nor98 scrapie circulating in Europe and the existence of strain mixtures in individual animals, strain typing in scrapie outbreaks is useful. To this end, a double animal bioassay in TgGoat-ARQ and TgBov mice seems to be the best method.

## Abbreviations

BSE: Bovine spongiform encephalopathy
CbCtx: Cerebellar cortex
CCtx: Cerebral cortex
COAL: Coalescing PrP^res^ deposits
dpi: Days post-inoculation
GLAS: Extracellular glia-associated PrP^res^ deposits
Hpth: Hypothalamus
Hpp: Hippocampus
IHC: Immunohistochemistry
ITGL: Intraglial PrP^res^ deposits
ITNR: Intraneuronal PrP^res^ deposits
kDa: Kilodalton
LINR: Linear PrP^res^ deposits
Midb: Midbrain
PET: Paraffin-embedded tissue
PLAQ: Vascular and non-vascular plaque PrP^res^ deposits
PoMe: Pons/medulla oblongata
PrP^C^: Cellular prion protein
PrP^res^: Proteinase K-resistant prion protein
PrP^Sc^: Disease abnormally misfolded prion protein
sCJD: Sporadic Creutzfeldt-Jakob disease
Stri: Corpus striatum
TE: Transmission efficiency
Thal: Thalamus
TgBov: BoTg-110
TgGoat-ARQ: OvTg-501
TSE: Transmissible spongiform encephalopathy
vCJD: Variant Creutzfeldt-Jakob disease
WB: Western blotting
